# Physical activity and movement quality among health sciences students: an exploratory cross-sectional study

**DOI:** 10.7717/peerj.21403

**Published:** 2026-06-05

**Authors:** Latifah Alenezi, Fareedah AlMohri, Fatma AlKandari, Sara Mullayousif, Nour AlMutairi, Ghala AlKhanfar, Atyab AlDubaikhi, Hind AlRujaib

**Affiliations:** 1 Physical Therapy Department, Faculty of Allied Health Sciences, Kuwait University, Kuwait City, Kuwait; 2 Ministry of Health, Kuwait City, Kuwait

**Keywords:** Physical activity, Movement quality, Functional movement screen, International physical activity questionnaire, Sedentary behavior, University students, Cross-sectional study, Kuwait

## Abstract

**Background:**

University students often sit for long periods while engaging in variable physical activity (PA). Whether higher PA relates to better movement quality is unclear in Middle East settings. This exploratory cross-sectional study examined associations between PA and Movement quality in health-sciences undergraduates and reported self-reported sitting time from the International Physical Activity Questionnaire (IPAQ) sitting item (not a validated sedentary-behavior measure).

**Methods:**

Students (*N* = 70; 58.6% female) completed the International Physical Activity Questionnaire–Short Form (IPAQ-SF; reported as Metabolic Equivalent of Task (MET) minutes per week) and an in-person Functional Movement Screen (FMS; seven tasks; total score 0–21). The primary model regressed FMS total on log-transformed PA, adjusting *a priori* for sex, Body Max Index (BMI), daily sitting hours, and academic major (Physical Therapy *vs* other). A secondary model examined the odds of FMS < 14. Exploratory component analyses used false discovery rate (FDR) control. Daily step counts from heterogeneous personal devices were treated as exploratory and excluded from primary models.

**Results:**

A total of 70 students were included in the analysis, of whom 55.7% scored below 14 on the FMS. The average physical activity level was 3,249.17 ± 2,425.61 MET-min/week, and the mean FMS score was 13.21 ± 2.21. In the adjusted linear model, physical activity was not significantly associated with total FMS score (*β* = 0.49, 95% CI [−0.18–1.16], *p* = 0.146). In the adjusted logistic model, physical activity was also not significantly associated with having an FMS score below 14 (OR = 0.61, 95% CI [0.31–1.21], *p* = 0.157). BMI showed a borderline, non-significant association with FMS < 14 (OR = 1.14, 95% CI [0.99–1.31], *p* = 0.072). In exploratory component-level analyses, no FMS component remained statistically significant after FDR correction.

**Conclusions:**

Physical activity was not significantly associated with movement quality after adjustment for sex, BMI, sitting time, and academic major. These findings suggest that physical activity alone may not independently explain variation in FMS scores in this sample.

## Introduction

University students occupy a pivotal developmental phase during which health behaviors consolidate and track into adulthood, shaping long-term cardiometabolic, musculoskeletal, cognitive, and psychosocial outcomes (Bull et al., 2020). University students often sit for long periods while engaging in variable physical activity (PA) (Castro et al., 2020). Their health is an area of particular concern (Castro et al., 2020; Aceijas et al., 2017). The 2020 World Health Organization (WHO) Guidelines recommend that adults accrue 150–300 min/week of moderate-intensity or 75–150 min/week of vigorous-intensity aerobic physical activity (PA) (or an equivalent combination), and, across all ages and abilities, reduce sedentary time because prolonged sitting is independently associated with adverse outcomes (Bull et al., 2020; World Health Organization, 2020). These recommendations provide a public-health frame for assessing and supporting movement behaviors in university settings (Bull et al., 2020; Castro et al., 2020).

A crucial conceptual distinction is that physical inactivity (not meeting PA guidelines) and sedentary behavior are not synonyms (Bull et al., 2020; World Health Organization, 2020). The Sedentary Behaviour Research Network (SBRN) defines sedentary behavior as any waking behavior in a sitting/reclining/lying posture with energy expenditure ≤ 1.5 METs (Tremblay et al., 2017). Treating inactivity and sedentary behavior as distinct constructs enhances measurement clarity, strengthens inference, and avoids misleading conclusions when a population is both insufficiently active and highly sedentary—a pattern frequently observed among university students (World Health Organization, 2020).

Evidence synthesised specifically for university populations indicates high sedentary time, often in the range of ∼7–11 h/day, with device-based estimates typically exceeding self-report and several reviews noting wide variability across studies (Moulin et al., 2021; Franco, Ferraz & Sousa, 2019). Such exposure levels are plausibly consequential given associations between prolonged sitting and cardiometabolic risk, mental health symptoms, and reduced fitness, with associations that persist even after accounting for physical activity (Xu et al., 2019; Wu et al., 2022; Dempsey et al., 2020). These findings justify parallel attention to increasing PA and reducing sitting, rather than treating them as a single behavior axis—especially because self-report tends to underestimate sedentary time by ∼1.7 h/day *versus* devices, which can mask the true burden in student cohorts (Prince et al., 2020).

Regional context matters. An overview of systematic reviews and meta-analyses covering 20 Middle East and North Africa (MENA) countries reported that, after 2000, only ∼51% of adults and ∼26% of youth were sufficiently active, with notable gender disparities and measurement inconsistencies for sedentary behavior prominent (Chaabane et al., 2020). The authors called for stronger surveillance and standardized instruments, issues directly relevant to student health in Gulf states, including Kuwait (Chaabane et al., 2020; Chaabane et al., 2021). Within Kuwait, studies among college students show a nuanced profile: high proportions self-classify as “highly active” by questionnaire, yet screen time remains high and sleep often insufficient, consistent with the conceptual independence of PA and sedentary time (Alkatan et al., 2021; Al-Haifi et al., 2023). Beyond how much students move or sit; movement quality offers a complementary lens on functional capacity relevant to daily living and pre-clinical impairments (Chaabane et al., 2021). Movement quality was assessed with a standardized field screen described in the Methods.

**Aim.** To investigate associations between IPAQ-Short PA and movement quality among health-sciences undergraduates, with self-reported sitting time described explicitly as the IPAQ sitting item (not a validated sedentary-behavior measure). We hypothesised that higher PA would be associated with higher movement quality scores after adjusting for sex, BMI, sitting time, and major, acknowledging the exploratory, non-causal nature of this cross-sectional design.

## Materials and Methods

### Study design

This exploratory, cross-sectional study was conducted among undergraduate students enrolled at the Health Sciences Centre (HSC) of Kuwait University. All participants provided written informed consent prior to data collection.

### Sample size

This study was designed as an exploratory cross-sectional investigation, and the sample size was determined based on the feasibility of conducting individual, in-person Functional Movement Screen (FMS) assessments, which are time- and resource-intensive. No formal *a priori* power analysis was performed. Therefore, we aimed to recruit approximately 70 participants, representing the maximum achievable number within the data-collection period. We acknowledge that the study was not powered to detect small associations, and the findings should be interpreted as preliminary, informing the design of larger prospective studies.

### Population and setting

Participants were undergraduate students, recruited from all faculties and departments of HSC, including Medicine, Pharmacy, Dentistry, and Allied Health Sciences (Physical Therapy, Occupational Therapy, Medical Laboratory Science, Radiological Sciences, and Health Informatics). Participants were selected through convenient sampling, encompassing students in all years of study. Inclusion criteria were enrolled student; age 18–26; ability to follow instructions during study period (*e.g.*, avoiding heavy exercise 24 h before the test). Exclusion criteria included current/recent musculoskeletal injury restricting movement; serious cardiopulmonary or neurological conditions; pregnancy. Screening was based on self-reported medical history and informal clinical indicators assessed by trained physiotherapists. The study was conducted in the Physical Therapy laboratory in the HSC Campus.

### Data collection

Ethical approval was obtained from the Institutional Review Boards of Kuwait University- Health Science Centre (Ref. 862/2024). All procedures were conducted in accordance with ethical standards and approved by the relevant institutional review board. Students were recruited *via* departmental e-mails, posted flyers, classroom announcements, and student group messaging. Recruitment was facilitated also through outreach efforts, including word-of-mouth, and social media. Participants signed an in-formed consent form to ensure their understanding of the purpose of this research study as well as the confidentiality of their responses. Upon obtaining informed consent, participants underwent a series of assessments conducted by trained physiotherapists. All assessments were completed in a single session following a structured protocol.

Participants first completed a demographic questionnaire (age, sex, height, weight, profession, and year of study), followed by self-reported scales, including the short form of International Physical Activity Questionnaire (IPAQ). Subsequently, two assessments were administered in the following order: REEDCO Posture Assessment and Functional Movement Screen (FMS). The entire assessment session lasted approximately 45–60 min per participant. All assessments were conducted by trained physiotherapists to ensure consistency and participant understanding. Data collection occurred over a four-month period from January to May 2024, commencing immediately after ethics approval.

### Data collection tools

Four components were used for data collection:

**Demographic Data Sheet:** This included 6 items covering age, sex, profession, year of study, height and weight. Height and weight were measured using standardised equipment; BMI =  kg/m^2^.

**Short Form of the International Physical Activity Questionnaire (IPAQ):** The short form of the International Physical Activity Questionnaire (IPAQ) was used to assess physical activity over the previous seven days (Craig et al., 2003). The IPAQ short form, administered either self-reported or *via* interviewer recording, has demonstrated good reliability and validity across diverse populations (Lee et al., 2011; Hagströmer, Oja & Sjöström, 2006). It captures time spent in vigorous activity, moderate activity, walking, and sitting.

Participants reported the frequency (days/week) and duration (minutes/day) of each activity type. Total physical activity was calculated in Excel by multiplying the time spent in each activity by its corresponding Metabolic Equivalent (MET) value and summing the resulting MET-minutes/week. Students were subsequently classified as having low, moderate, or high activity levels according to IPAQ scoring protocol. A trained physiotherapist administered the questionnaire to enhance reliability, ensure correct interpretation of the items, and support students during the reporting process.

To complement self-reported measures, students who owned a smartwatch, pedometer, or smartphone with step-tracking features provided their average step count for the past seven days.

**Functional Movement Screen (FMS):** It was used to assess functional movement quality. The FMS is a validated screening tool composed of seven movement tasks scored from 0 to 3, providing both individual item scores and a total score out of 21. Higher scores indicate better functional mobility and stability, whereas lower scores reflect compensations, mobility limitations, or pain. A score of 0 is assigned when the movement elicits pain; 1 indicates inability to perform the task; 2 reflects completion with compensations; and 3 represents optimal, pain-free movement performance. FMS demonstrates moderate to good inter- and intra-rater reliability across raters and contexts (Moran, Schneiders & Sullivan, 2016; Teyhen et al., 2012; Morgan et al., 2023; Bonazza et al., 2017). In this study, the total FMS score was treated strictly as a movement quality outcome rather than a diagnostic measure; not interpreting FMS cut-offs as injury risk (Cook et al., 2014). Any reference to “injury risk” reflects FMS-defined threshold conventions only, acknowledging the ongoing debate regarding the predictive validity of the commonly cited ≤14 cut-off outside athletic populations (Moran et al., 2017; Pollen, Keitt & Trojian, 2021).

The screening was administered by a trained physiotherapist to ensure standardized instructions and consistent scoring. All assessments were conducted by the same examiner and at the same time of day to reduce variability. Participants were given clear instructions before each task and performed a brief warm-up; they were advised to avoid vigorous exercise for 24 h prior to testing. The seven movement tasks included: deep squat, hurdle step, in-line lunge, shoulder mobility, active straight leg raise, trunk stability push-up, and rotary stability. These tasks collectively evaluate mobility, stability, balance, and movement asymmetry, helping identify movement dysfunctions that may increase injury risk.

**Optional Step Count (Exploratory Measure):** An optional exploratory measure was included in which participants could provide their 7-day average daily step count obtained from personal devices (*e.g.*, smartphones, smartwatches, or pedometers). Because device types, algorithms, and wear-time patterns vary substantially, step count was treated as an exploratory variable rather than a primary exposure (Höchsmann et al., 2020; Adamakis, 2023; Johnston et al., 2021). Interpretation relied only on population-level step-defined indices, specifically, <5,000 steps/day as sedentary and ≥10,000 steps/day as active, as established in step-classification literature (Tudor Locke et al., 2013; Ainsworth, Rivière & Florez Pregonero, 2023).

### Variables

The primary exposure was physical activity measured by the IPAQ-short, expressed as (1) MET-min/week (continuous, log-transformed for regression) and (2) IPAQ PA categories (low, moderate, high; treated as an ordered variable). Self-reported sitting time (minutes/day from the IPAQ sitting item) was included as a covariate rather than a validated sedentary-behavior measure. The primary outcome was the Functional Movement Screen (FMS) total score (0–21). A secondary dichotomous outcome (FMS < 14) was derived as an FMS-defined threshold without implying injury prediction. Exploratory outcomes were the seven individual FMS component scores. *A priori* covariates included sex, BMI (kg/m^2^), daily sitting time, and academic major (PT *vs* other). Daily step counts from heterogeneous personal devices were treated as exploratory only, not included in primary regression models due to device-dependent variability in accuracy and wear-time.

### Bias mitigation/quality control

To minimize measurement bias, all FMS assessments were performed by a single trained assessor using standardized instructions and scoring procedures, consistent with published FMS reliability evidence. The IPAQ-short was administered using the official script to reduce recall variability; however, we acknowledge that self-report PA may overestimate true activity levels relative to device-based measures. Following SBRN guidance, the IPAQ sitting item was interpreted strictly as self-reported sitting time, not as a validated sedentary-behavior measure. To reduce confounding, all primary and secondary models were adjusted for sex, BMI, sitting hours, and academic major. Selection bias was limited by inviting all eligible students during the recruitment period, though the over-representation of PT students is noted and addressed analytically. Multiplicity bias in component-level analyses was controlled using false discovery rate (FDR) procedures.

### Statistical analysis

The primary model specified FMS total as outcome with log-transformed PA (MET-min/week) as predictor, adjusting for sex (Female), BMI (kg/m^2^), daily sitting hours (IPAQ item), and academic major (PT *vs* other). A secondary logistic regression model examined the odds of FMS < 14 using the same covariates. For exploratory outcomes, each FMS component was modeled similarly; false-discovery rate (FDR) was used to control multiplicity across seven components. We reported β coefficients (95% CIs) and odds ratios (95% CIs); all tests were two-sided with *α* = 0.05.

**Figure 1:**
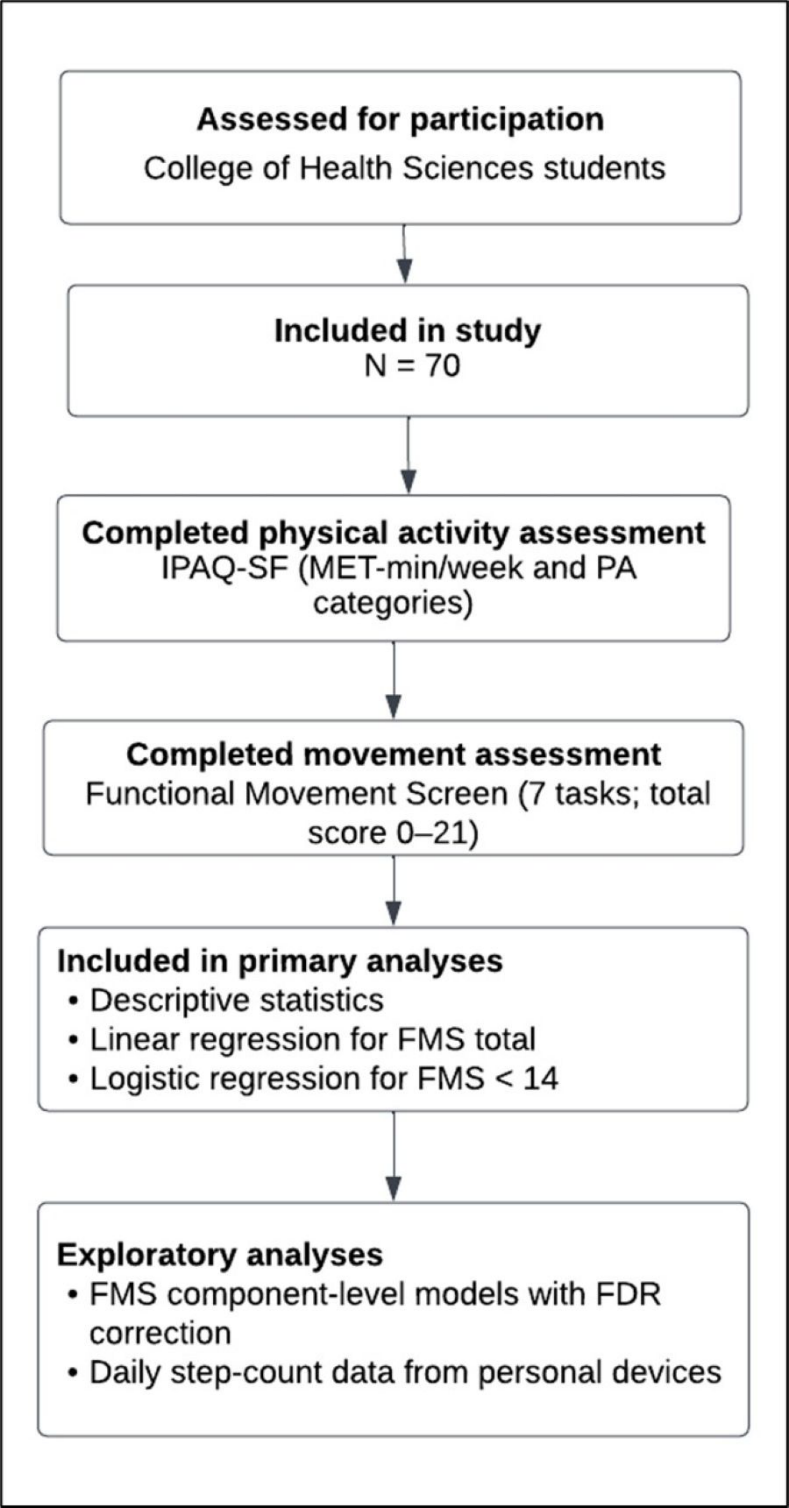
Participant flow and analytic framework of the study.

Figure 1 shows the participant flow and analytic framework of the study. All 70 included participants completed both the IPAQ-SF and the in-person FMS assessment. The primary analyses examined the association between physical activity and movement quality using adjusted multivariable models, while component-level FMS analyses and device-based step counts were treated as exploratory.

## Results

### Sample characteristics

As shown in Table 1, the mean age of the participants was 20.6 ± 1.3 years, and more than half of the sample were female (41, 58.6%). The average height and weight were 165 cm and 66 kg, respectively, while the mean BMI was 24.1 ± 4.3 kg/m^2^. In terms of lifestyle, participants took an average of 5,369 ± 2,750 steps per day and spent about 7.13 ± 1.99 h sitting each day, which suggests a fairly sedentary pattern. At the same time, the mean physical activity level was 3,249.17 ± 2,425.61 MET-min/week, showing that activity levels varied considerably across participants. The mean FMS total score was 13.21 ± 2.21, with scores ranging from 8 to 19. More than half of the participants (39, 55.7%) had an FMS score below 14.

**Table 1:** Baseline characteristics of the sample (*N* = 70).

Characteristic	Value
Age, years	20.6 ± 1.3
Female, n (%)	41 (58.6%)
Height, cm	165
Weight, kg	66
BMI, kg/m^2^	24.1 ± 4.3
Steps/day	5,369 ± 2,750
Sitting time, h/day	7.13 ± 1.99
PAQ MET-min/week	3,249.17 ± 2,425.61
FMS total (0–21)	13.21 ± 2.21 (min 8, max 19)
FMS <14, n (%)	39 (55.7%)

### Association between PA and FMS (primary/secondary models)

A multivariable analysis was conducted to examine the association between physical activity and movement quality after adjusting for sex, BMI, sitting time, and academic major (Table 2). In Model A, where FMS total score was treated as a continuous outcome, physical activity was not significantly associated with FMS score after adjustment (*β* = 0.49, 95% CI [−0.18–1.16], *p* = 0.146). None of the covariates showed a statistically significant association with total FMS score.

**Table 2:** Multivariable models for movement quality.

Predictor	*β*/OR	95% CI	SE/—	*p*-value
Model A (Linear): FMS total ∼log-PA + covariates				
Log (PA, MET-min/week)	+0.49	(−0.18, +1.16)	0.33	0.146
Female (*vs* male)	−0.95	(−2.21, +0.31)	0.63	0.137
BMI (kg/m^2^)	−0.07	(−0.19, +0.06)	0.06	0.295
Sitting time (h/day)	+0.08	(−0.19, +0.35)	0.13	0.561
PT major (*vs* other)	+0.23	(−0.89, +1.35)	0.56	0.685
Model B (Logistic): FMS<14 ∼log-PA + covariates				
log (PA, MET-min/week)	0.61	(0.31, 1.21)	–	0.157
Female (*vs* male)	1.80	(0.53, 6.15)	–	0.350
BMI (kg/m^2^)	1.14	(0.99, 1.31)	–	0.072
Sitting time (h/day)	0.92	(0.70, 1.21)	–	0.530
PT major (*vs* other)	1.32	(0.44, 4.01)	–	0.622

**Notes.**

*β* regression coefficient OR odds ratio CI confidence interval

All models adjusted for sex, BMI, sitting hours, and major.

In Model B, where the outcome was having an FMS score below 14, physical activity also did not show a statistically significant association after adjustment (OR = 0.61, 95% CI [0.31–1.21], *p* = 0.157). BMI showed a borderline, non-significant association with the odds of scoring below 14 (OR = 1.14, 95% CI [0.99–1.31], *p* = 0.072), while the other covariates were not statistically significant.

Additional exploratory analyses were performed for the seven individual FMS components using adjusted models with false discovery rate (FDR) correction. While a few associations appeared significant before correction, none remained statistically significant after FDR adjustment (all *q* ≥ 0.165; Table 3).

**Table 3:** Component-level associations with log (PA) adjusted for covariates (FDR-controlled).

FMS component	*β* (log-PA)	SE	*p*	q (FDR)
Deep Squat	+0.21	0.12	0.079	0.184
Hurdle Step	+0.05	0.10	0.612	0.985
In-line lunge	−0.00	0.12	0.997	0.997
Shoulder mobility	−0.05	0.13	0.704	0.985
Active SLR	+0.10	0.04	0.024	0.165
Push-Up	+0.18	0.10	0.074	0.184
Rotary stability	+0.00	0.09	0.972	0.997

## Discussion

This cross-sectional study set out to examine whether physical activity (PA), assessed with the IPAQ-short, is associated with movement quality as measured by the Functional Movement Screen (FMS) among health-sciences undergraduates. We also recorded self-reported sitting time (IPAQ sitting item) and explored FMS component scores. In unadjusted analyses, higher PA was weakly but significantly associated with higher FMS scores. However, in covariate-adjusted models (sex, BMI, sitting hours, major) the association was not statistically significant, and no FMS component remained significant after false-discovery rate (FDR) correction. Males had higher unadjusted FMS, steps, and PA than females. To our knowledge, this is the first report from Kuwait to analyse PA–FMS associations in a student cohort using an *a priori* multivariable framework with multiplicity control, thereby providing context-specific baseline evidence and an analytic template for future work in the region.

### How our findings fit within student movement and sitting patterns

International guidance recognizes that PA confers broad health benefits while sedentary time should be reduced, independent of exercise dose (Bull et al., 2020; World Health Organization, 2020). Crucially, physical inactivity and sedentary behavior are distinct constructs; the latter is defined by the Sedentary Behaviour Research Network (SBRN) as ≤1.5 METs in a sitting/reclining posture (Tremblay et al., 2017). Among university students, a large meta-analysis estimated ∼7.3 h/day of sedentary time by self-report and ∼9.8 h/day by devices, with evidence of an upward trend over the last decade (Castro et al., 2020). Our cohort’s mean sitting time (∼7.1 h/day) aligns closely with these global figures. At the same time, MENA-wide syntheses report substantial proportions of adults and youth not meeting PA guidelines, with gender disparities and measurement heterogeneity (Chaabane et al., 2020). Kuwaiti student data similarly indicate that some cohorts self-classify as highly active while still accruing considerable screen/sitting time (Alkatan et al., 2021; Al-Haifi et al., 2023). This “active-yet-sedentary” pattern helps explain why our unadjusted PA–FMS correlation was detectable but attenuated to non-significance after accounting for sex, BMI, sitting hours, and major-behaviors and characteristics that covary strongly in student life.

### Interpreting the PA–FMS association in light of measurement and confounding

Our unadjusted correlation (*ρ* ≈0.26–0.30) is consistent with a small association between movement quantity (PA) and movement quality (FMS). Yang et al. (2024) reported similar positive cross-sectional relationships between PA and FMS in Chinese college students, where higher PA was significantly associated with higher FMS scores and lower BMI (Yang et al., 2024). The IPAQ-short shows acceptable reliability/validity across 12 countries but only moderate correlations with accelerometry (Craig et al., 2003); non-differential error in self-reported PA will bias associations toward the null. Moreover, adjusting for sex, BMI, and sitting hours is essential in student cohorts, where these factors are correlated with both PA and movement performance. In our data, this careful adjustment rendered the PA–FMS association non-significant, emphasizing that confounding and measurement imprecision can easily mask small true relationships in samples of this size. These findings echo broader student research showing that device-based sitting time and nuanced, domain-specific PA often sharpen estimates compared with a single global self-report instrument (Castro et al., 2020).

### Component-level findings and the question of “which movement qualities track with PA?”

At the task level, we observed nominal (uncorrected) associations for Active Straight-Leg Raise, Deep Squat, and Push-Up, but none survived FDR correction across seven FMS components. This pattern is plausible: the FMS composites integrate multiple domains (mobility, stability, symmetry), and modest weekly differences in PA—especially if dominated by walking reported on the IPAQ—may not consistently translate into task-specific gains once we control for sex, BMI, sitting, and major. Recent reviews of movement-screening tools argue that single-score or single-test predictions are unlikely to generalize across populations; multifactorial models that integrate workload, fitness, prior injury, and lifestyle perform better for risk stratification and change detection (Bonazza et al., 2017; Moran et al., 2017; Eckart et al., 2025). From a mechanistic standpoint, trunk stability and hip mobility tasks may respond to specific strengthening or mobility programming rather than to general increases in PA minutes—underscoring the importance of targeted exercise when the goal is to improve identifiable movement deficits.

### Gender differences and regional context

We observed higher FMS, steps, and PA among male students, mirroring regional reports from Kuwait and the wider MENA context (Chaabane et al., 2020; Alkatan et al., 2021; Al-Haifi et al., 2023). Cultural, environmental, and curricular factors can constrain activity opportunities for women; climatic conditions also promote car-based transport and indoor study time. These gender differentials may interact with body composition, training history, and confidence in motor tasks, creating compound advantages for males in push-up and squat patterns even when the absolute PA dose is modest. Such patterns illustrate why sex must be retained as a covariate and also justify sex-sensitive campus strategies to increase opportunities for strength, balance, and mobility training in women alongside PA promotion.

### Framing FMS appropriately: reliable movement quality, debated predictor of injury

The FMS has moderate-to-good reliability across raters and contexts (Teyhen et al., 2012), making it useful for educational and screening purposes in non-athletic populations. However, meta-analyses and systematic reviews caution that the FMS composite (especially the ≤14 cut-off) is not a stand-alone injury-prediction tool outside select athlete or military cohorts (Bonazza et al., 2017; Moran et al., 2017; Philp et al., 2018). Consistent with this literature, we have framed FMS<14 strictly as an FMS-defined threshold and avoided causal claims. Positioning the FMS as a movement-quality outcome—rather than as a prognostic device—better reflects its strengths, encourages corrective exercise programming for identified deficits, and avoids over-promising predictive utility in general student samples.

### Implications (research, practice, education)

For research, our findings argue for multimodal measurement in students: combine device-based PA and sedentary time with validated functional tests, and pre-specify multivariable models that adjust for sex, BMI, sitting, and academic major. For practice, campus wellness should move beyond aerobic minutes to include brief, embedded neuromotor sessions (mobility, trunk stability, balance) in long lectures and labs, interventions that target the specific qualities FMS interrogates. For university health promotion and curriculum design, movement quality should be considered alongside PA volume. Embedding brief, varied movement practice during lectures and labs (*e.g.*, mobility sequences, trunk stability tasks) can complement minutes-based PA targets and may benefit neuromuscular control—consistent with WHO’s “every move count” message. Simultaneously, initiatives should directly target sitting reduction (*e.g.*, active breaks, standing options, time-based prompts), because students can be physically active and highly sedentary at the same time. For education, integrating the FMS as a formative tool can help students observe, correct, and re-test movement patterns; however, staff and students should be explicitly trained not to interpret FMS as a predictive risk test in this population.

### Recommendations/future directions

Prospective studies with larger, more diverse student samples should evaluate whether device-measured PA and posture-coded sedentary time (inertial sensors/accelerometers) predict longitudinal changes in FMS and academic outcomes. Trials that compare generic PA promotion to targeted neuromotor programs (*e.g.*, short stability/mobility curricula integrated into classes) could clarify which components of movement quality are most modifiable, and in whom. Multifactorial models that incorporate workload, prior pain/injury, sleep, and psychosocial variables should be tested, consistent with contemporary screening frameworks.

### Limitations

This study is cross-sectional and cannot support causal inference. Self-reported IPAQ-short introduces recall/social-desirability bias, and the sitting item is not a validated sedentary-behavior measure under SBRN; both issues likely attenuate associations. The sample is a convenience cohort from a single health-sciences centre with an over-representation of PT majors, which may limit generalizability; we adjusted for major in all models, but residual confounding is possible. Step-count data came from heterogeneous personal devices with unknown wear-time, so they were treated as exploratory. Finally, our component-level analyses required FDR correction; none remained significant, which reduces the risk of false positives but may also obscure small true effects in a sample of this size.

### Conclusion

In summary, unadjusted analyses suggested that greater PA is associated with better movement quality (FMS) in health-sciences students, but this association was not significant after accounting for sex, BMI, sitting hours, and academic major; and no FMS components survived multiplicity control. These results support a nuanced approach to student movement health: address PA and sitting as distinct behaviors, use FMS formatively to guide corrective exercise, and prioritize device-based and prospective designs to clarify causal pathways.

##  Supplemental Information

10.7717/peerj.21403/supp-1 Supplemental Information 1 ExRaw data Anonymous Raw data containing all participants demographics and scores in the outcome measures used in study

10.7717/peerj.21403/supp-2 Supplemental Information 2 FMS Data Codebook Codebook representing the coded data in more details

## Additional Information and Declarations

### Data Availability

The following information was supplied regarding data availability: The raw measurements are provided in Supplemental Files 1. These files include all participants sociodemographic, Level of Physical activity measured by IPAQ, and the functional movement patterns using FMS assessment. This subset of participants was included in the statistical analysis to find association between physical activity and functional performance.
