# Supplementary material for: Physical activity and movement quality among health sciences students: an exploratory cross-sectional study
Source: PeerJ. 2026 Jun 5;14:e21403. doi: 10.7717/peerj.21403 (PMC13245423; doi:10.7717/peerj.21403)
Supplement: Supplemental Information 2 — Codebook representing the coded data in more details [file peerj-14-21403-s002.docx]

**FMS data codebook:**

| **variable_name** | **variable_label** | **code** | **label** | **type** |
| --- | --- | --- | --- | --- |
| Gender | Gender of participant | 1 | Male | nominal |
| Gender | Gender of participant | 2 | Female | nominal |
| Year_of_study | Year of study | 1 | Year 1 | ordinal |
| Year_of_study | Year of study | 2 | Year 2 | ordinal |
| Year_of_study | Year of study | 3 | Year 3 | ordinal |
| Year_of_study | Year of study | 4 | Year 4 | ordinal |
| BMI_Category_Coded | BMI category (coded) | 1 | Underweight | ordinal |
| BMI_Category_Coded | BMI category (coded) | 2 | Normal | ordinal |
| BMI_Category_Coded | BMI category (coded) | 3 | Overweight | ordinal |
| BMI_Category_Coded | BMI category (coded) | 4 | Obese | ordinal |
| PA_Category_Coded | Physical activity category (coded) | 1 | Low | ordinal |
| PA_Category_Coded | Physical activity category (coded) | 2 | Moderate | ordinal |
| PA_Category_Coded | Physical activity category (coded) | 3 | High | ordinal |
| Deep_Squat | Deep Squat (FMS item score) | 0 | Pain with movement / unable due to pain | ordinal |
| Deep_Squat | Deep Squat (FMS item score) | 1 | Poor pattern | ordinal |
| Deep_Squat | Deep Squat (FMS item score) | 2 | Compensated pattern | ordinal |
| Deep_Squat | Deep Squat (FMS item score) | 3 | Performs correctly | ordinal |
| Hurdle_Step | Hurdle Step (FMS item score) | 0 | Pain with movement / unable due to pain | ordinal |
| Hurdle_Step | Hurdle Step (FMS item score) | 1 | Poor pattern | ordinal |
| Hurdle_Step | Hurdle Step (FMS item score) | 2 | Compensated pattern | ordinal |
| Hurdle_Step | Hurdle Step (FMS item score) | 3 | Performs correctly | ordinal |
| In_line_lunge | In line lunge (FMS item score) | 0 | Pain with movement / unable due to pain | ordinal |
| In_line_lunge | In line lunge (FMS item score) | 1 | Poor pattern | ordinal |
| In_line_lunge | In line lunge (FMS item score) | 2 | Compensated pattern | ordinal |
| In_line_lunge | In line lunge (FMS item score) | 3 | Performs correctly | ordinal |
| Shoulder_mobility | Shoulder mobility (FMS item score) | 0 | Pain with movement / unable due to pain | ordinal |
| Shoulder_mobility | Shoulder mobility (FMS item score) | 1 | Poor pattern | ordinal |
| Shoulder_mobility | Shoulder mobility (FMS item score) | 2 | Compensated pattern | ordinal |
| Shoulder_mobility | Shoulder mobility (FMS item score) | 3 | Performs correctly | ordinal |
| Active_SLR | Active SLR (FMS item score) | 0 | Pain with movement / unable due to pain | ordinal |
| Active_SLR | Active SLR (FMS item score) | 1 | Poor pattern | ordinal |
| Active_SLR | Active SLR (FMS item score) | 2 | Compensated pattern | ordinal |
| Active_SLR | Active SLR (FMS item score) | 3 | Performs correctly | ordinal |
| Push_Up | Push Up (FMS item score) | 0 | Pain with movement / unable due to pain | ordinal |
| Push_Up | Push Up (FMS item score) | 1 | Poor pattern | ordinal |
| Push_Up | Push Up (FMS item score) | 2 | Compensated pattern | ordinal |
| Push_Up | Push Up (FMS item score) | 3 | Performs correctly | ordinal |
| Rotatory_Stability | Rotatory Stability (FMS item score) | 0 | Pain with movement / unable due to pain | ordinal |
| Rotatory_Stability | Rotatory Stability (FMS item score) | 1 | Poor pattern | ordinal |
| Rotatory_Stability | Rotatory Stability (FMS item score) | 2 | Compensated pattern | ordinal |
| Rotatory_Stability | Rotatory Stability (FMS item score) | 3 | Performs correctly | ordinal |
| Posture_head | Posture head | 1 | Poor | ordinal |
| Posture_head | Posture head | 2 | Fair | ordinal |
| Posture_head | Posture head | 3 | Good | ordinal |
| Posture_shoulder | Posture shoulder | 1 | Poor | ordinal |
| Posture_shoulder | Posture shoulder | 2 | Fair | ordinal |
| Posture_shoulder | Posture shoulder | 3 | Good | ordinal |
| Spine_Posture | Spine Posture | 1 | Poor | ordinal |
| Spine_Posture | Spine Posture | 2 | Fair | ordinal |
| Spine_Posture | Spine Posture | 3 | Good | ordinal |
| Posture_hips | Posture hips | 1 | Poor | ordinal |
| Posture_hips | Posture hips | 2 | Fair | ordinal |
| Posture_hips | Posture hips | 3 | Good | ordinal |
| Kyphosis | Kyphosis | 0 | Absent | binary |
| Kyphosis | Kyphosis | 1 | Present | binary |
| Flat_Foot | Flat Foot | 0 | Absent | binary |
| Flat_Foot | Flat Foot | 1 | Present | binary |
| High_Arch | High Arch | 0 | Absent | binary |
| High_Arch | High Arch | 1 | Present | binary |
| Hypermobility | Hypermobility | 0 | Absent | binary |
| Hypermobility | Hypermobility | 1 | Present | binary |
| Rounded_Shoulder | Rounded Shoulder | 0 | Absent | binary |
| Rounded_Shoulder | Rounded Shoulder | 1 | Present | binary |
| Ant_Pelvic_Tilt | Ant Pelvic Tilt | 0 | Absent | binary |
| Ant_Pelvic_Tilt | Ant Pelvic Tilt | 1 | Present | binary |
| Scoliosis | Scoliosis | 0 | Absent | binary |
| Scoliosis | Scoliosis | 1 | Present | binary |
| Lordosis | Lordosis | 0 | Absent | binary |
| Lordosis | Lordosis | 1 | Present | binary |
| Forward_Head | Forward Head | 0 | Absent | binary |
| Forward_Head | Forward Head | 1 | Present | binary |
| Hypomobility | Hypomobility | 0 | Absent | binary |
| Hypomobility | Hypomobility | 1 | Present | binary |
| Genu_Varum | Genu Varum | 0 | Absent | binary |
| Genu_Varum | Genu Varum | 1 | Present | binary |
| Hip_Issue | Hip Issue | 0 | Absent | binary |
| Hip_Issue | Hip Issue | 1 | Present | binary |
| PCL_Issue | PCL Issue | 0 | Absent | binary |
| PCL_Issue | PCL Issue | 1 | Present | binary |
| Deformity_Coded | Any deformity present (coded) | 0 | No deformity | binary |
| Deformity_Coded | Any deformity present (coded) | 1 | Some deformity present | binary |
| None | No musculoskeletal issue indicator | 0 | Issue present (not none) | binary |
| None | No musculoskeletal issue indicator | 1 | No issues (none) | binary |
| Pain_level | Pain level (0–10) | 0 | No pain | ordinal |
| Pain_level | Pain level (0–10) | 10 | Worst imaginable pain | ordinal |
